# Supplementary figures and images for: Bivalve microbiomes are shaped by host species, size, parasite infection, and environment
Source: PeerJ. 2024 Oct 8;12:e18082. doi: 10.7717/peerj.18082 (PMC11468899; doi:10.7717/peerj.18082)

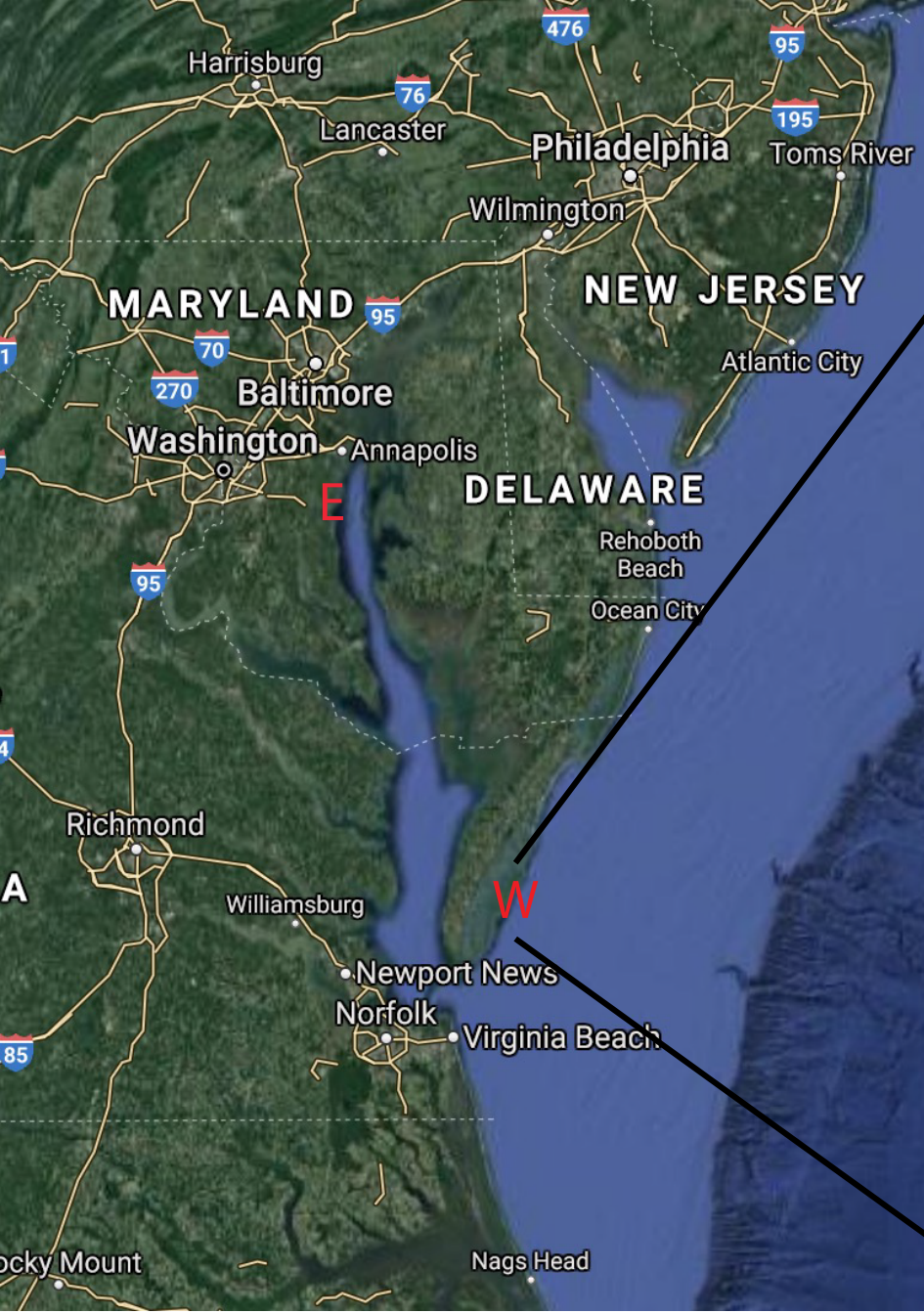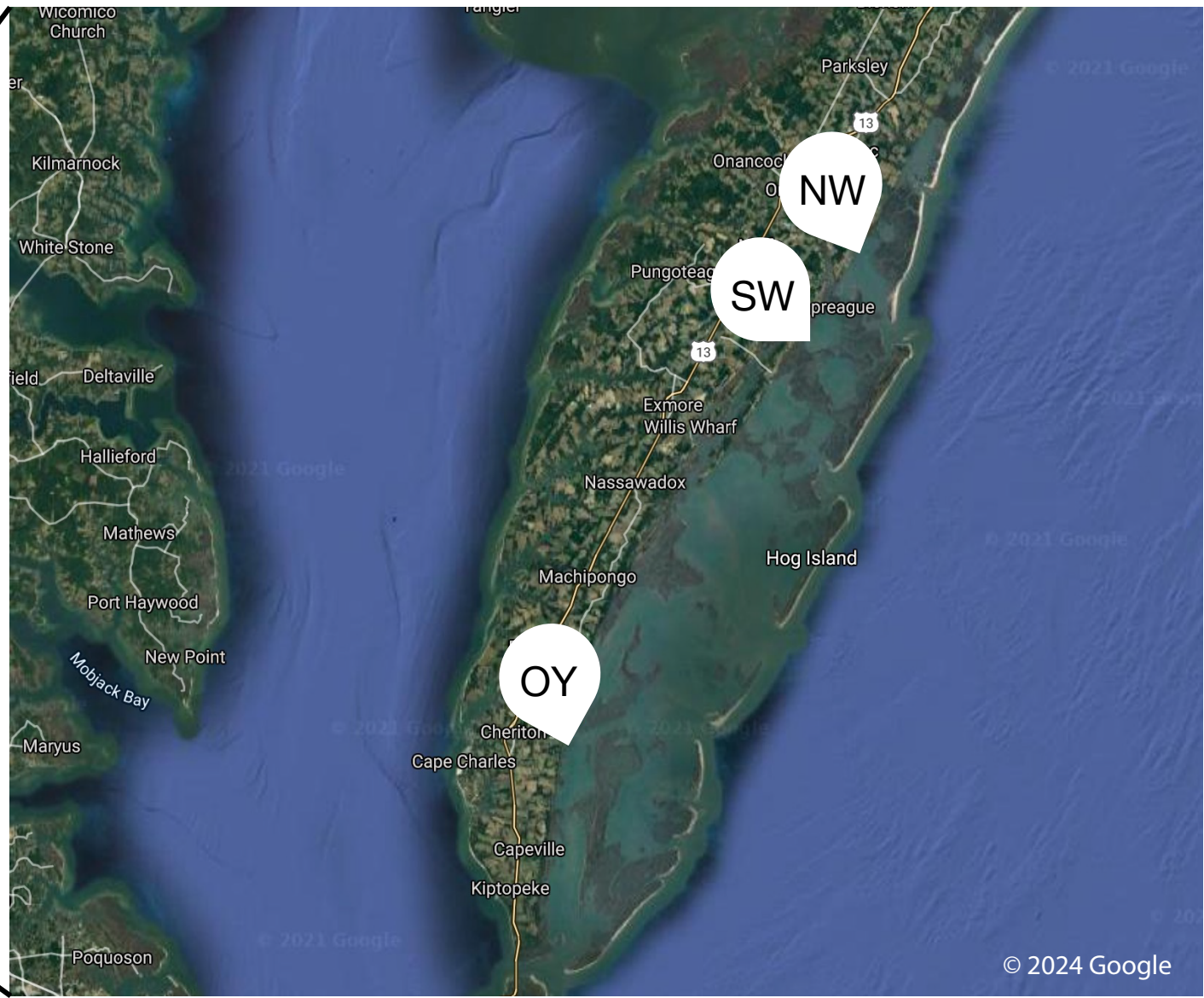

Supplement: Supplemental Information 1 — Edgewater (2018 experiment location) is marked with an E, while Wachapreague (2017 experiment location) is marked with a W. The inset shows the specific locations of the three sites around Wachapreague, VA. Map data © 2024 Google. [file peerj-12-18082-s001.pdf]

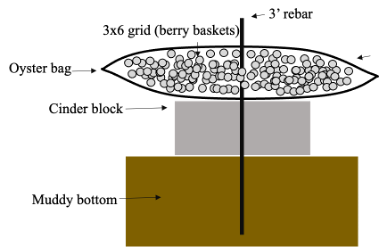

Supplement: Supplemental Information 2 — How oyster bags were affixed to the bottom in the 2017 experiment. [file peerj-12-18082-s002.png]

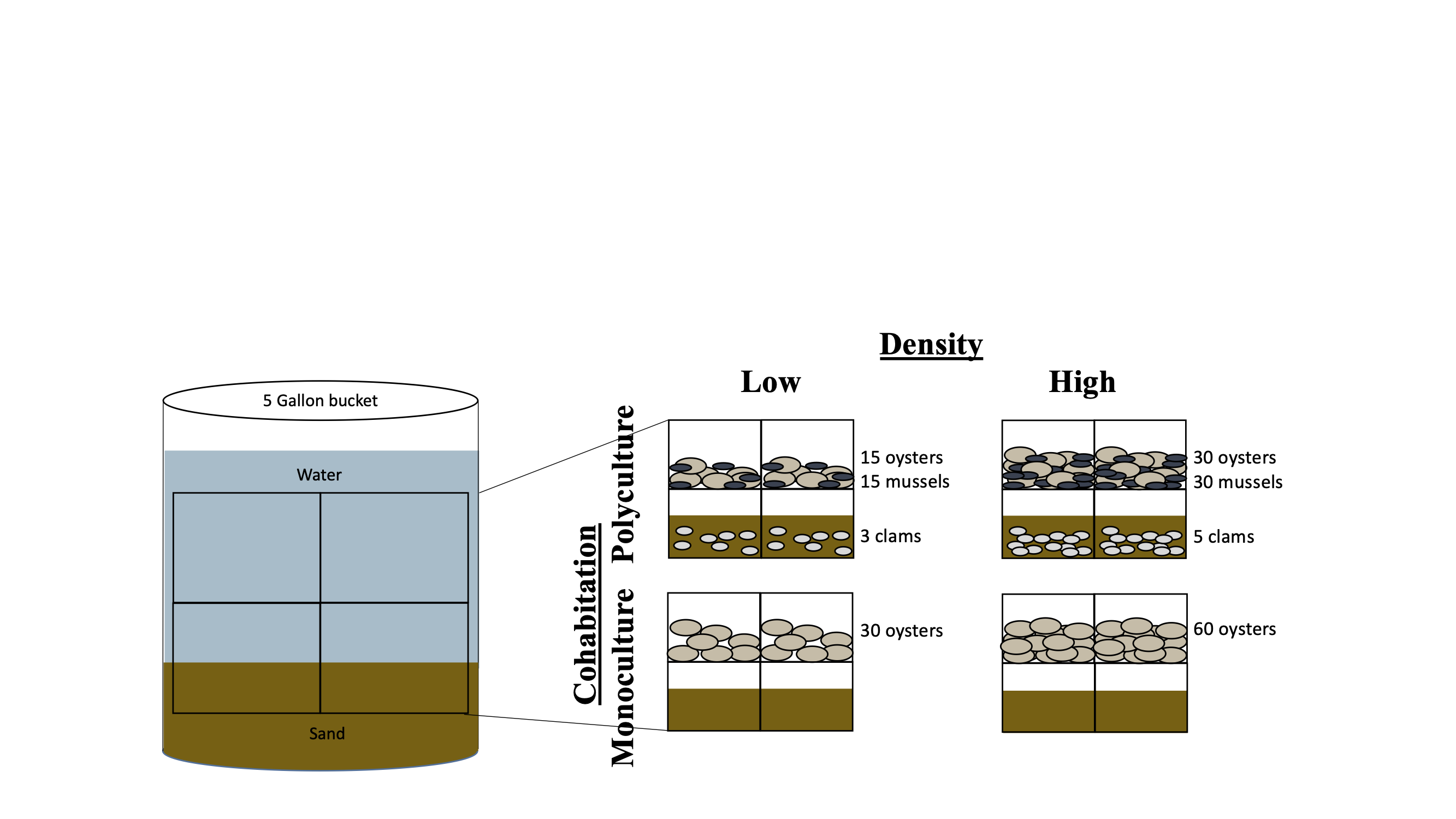

Supplement: Supplemental Information 3 — The inside of an experimental mesocosm (bucket) as well as different treatment levels. [file peerj-12-18082-s003.png]

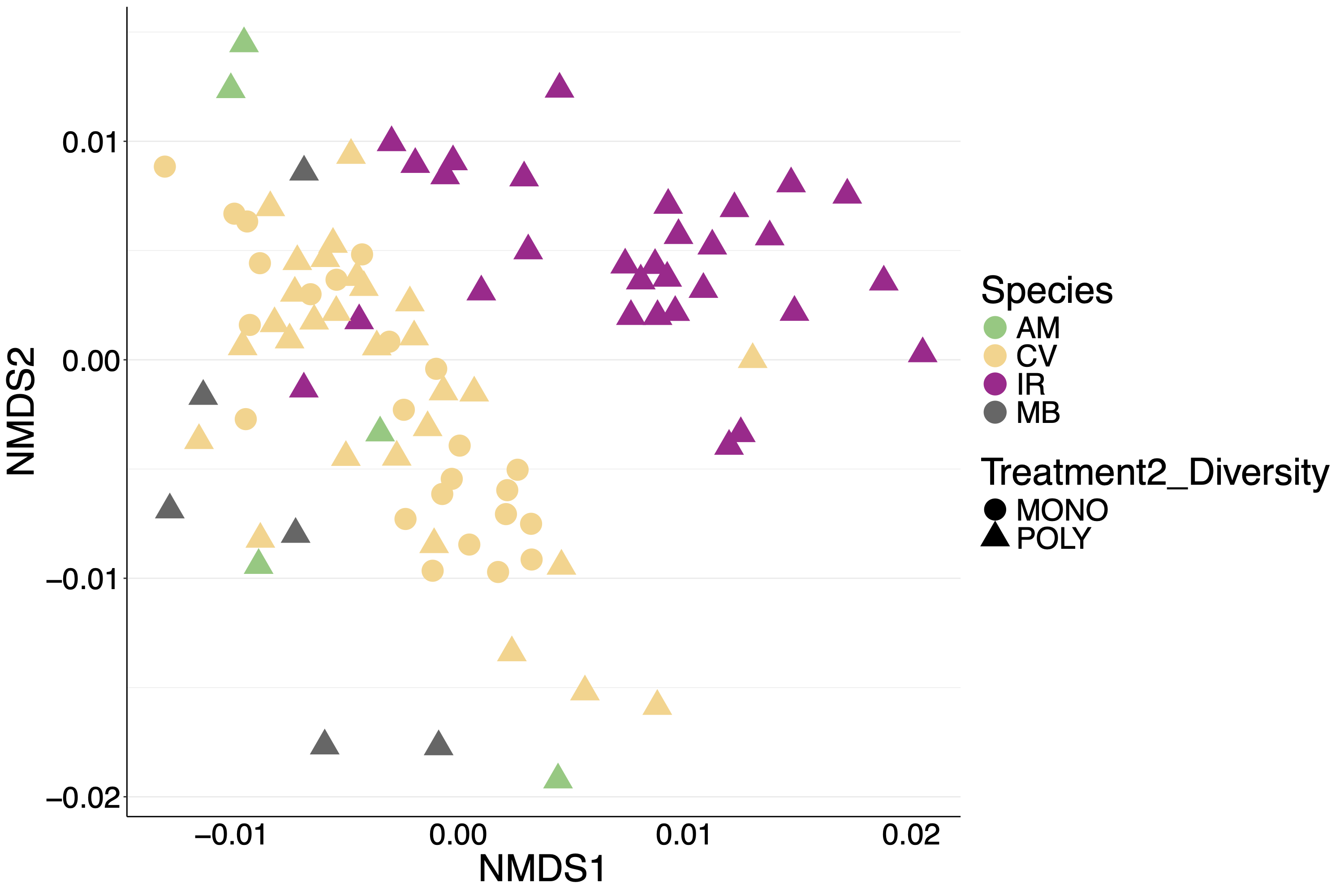

Supplement: Supplemental Information 4 — Individuals are color coded by species with symbols indicating experimental treatments. nMDS converged after 20 permutations, stress=0.19. [file peerj-12-18082-s004.png]

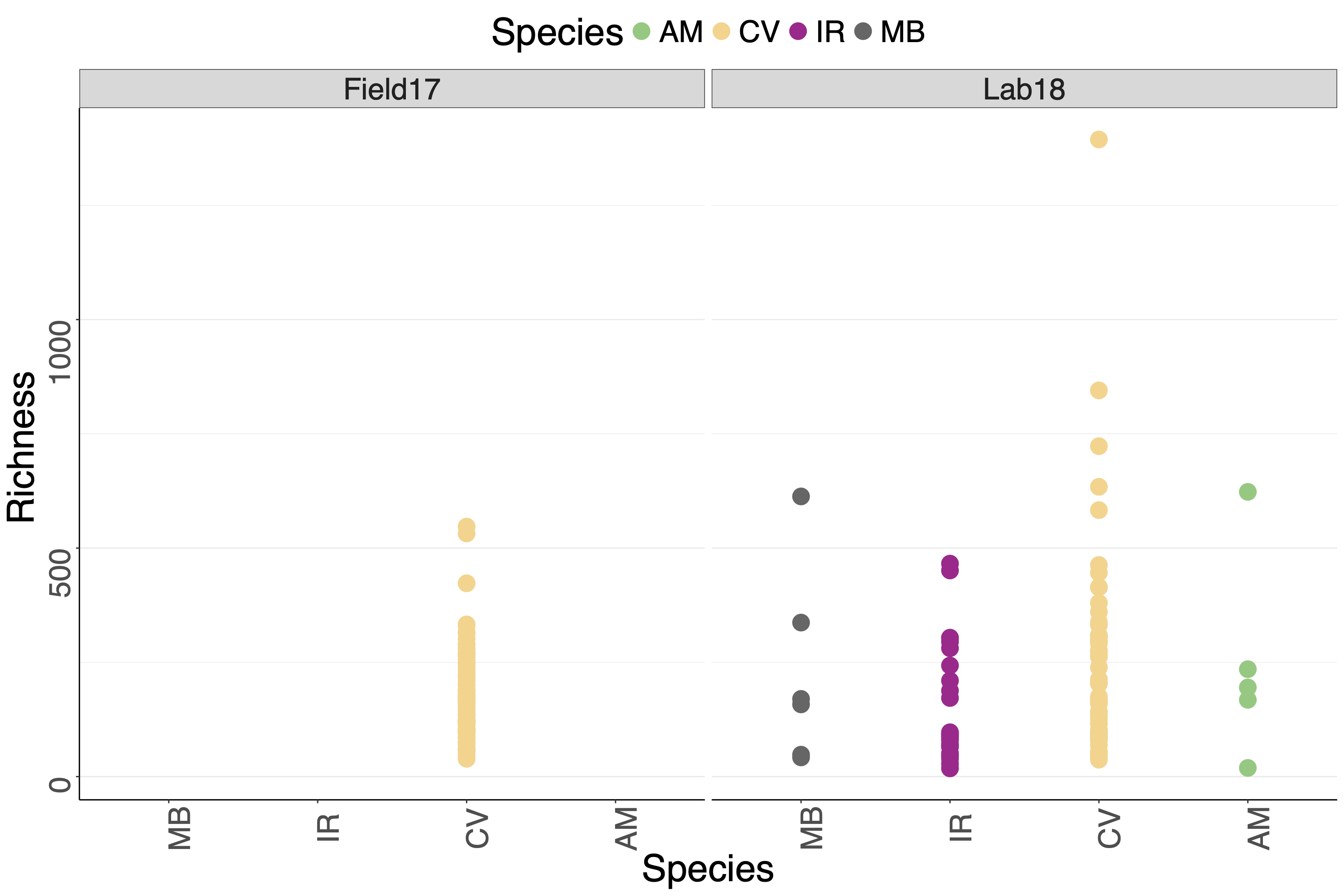

Supplement: Supplemental Information 5 — Richness is calculated as observed number of ASVs. [file peerj-12-18082-s005.png]

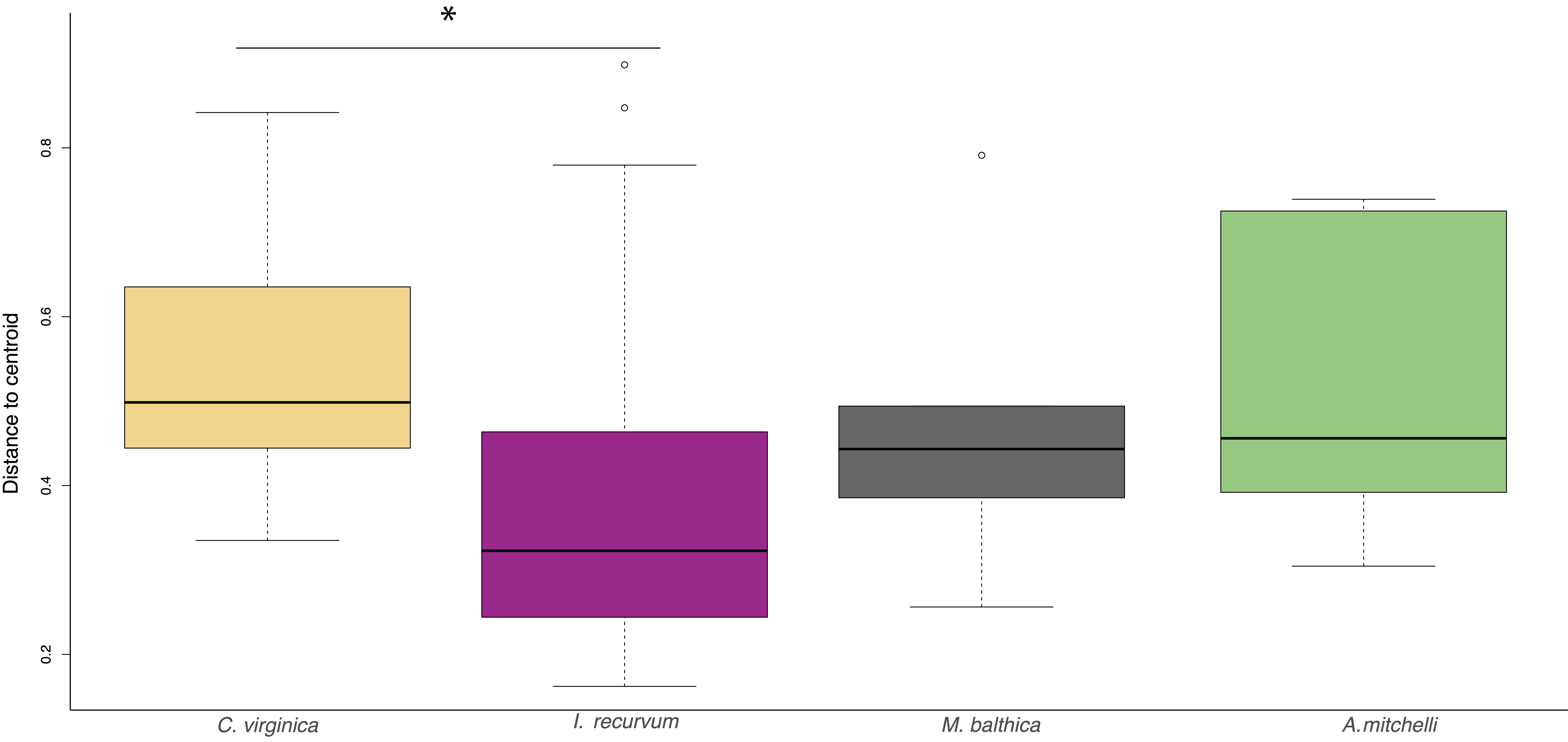

Supplement: Supplemental Information 6 — Dispersion measured as distance to centroid for each group. Stars indicate significantly different values. [file peerj-12-18082-s006.png]

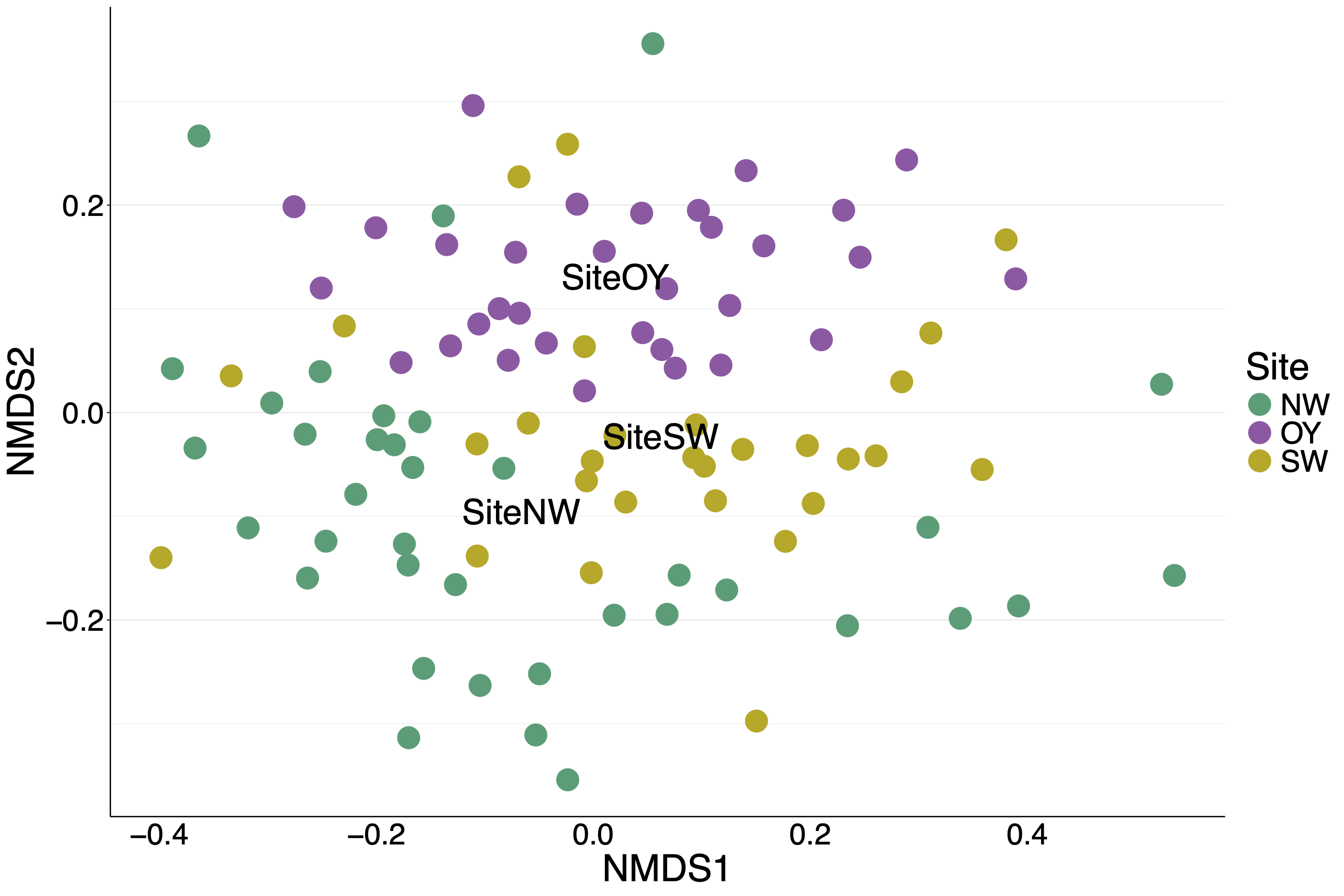

Supplement: Supplemental Information 7 [file peerj-12-18082-s007.png]
