# Supplementary material for: Bivalve microbiomes are shaped by host species, size, parasite infection, and environment
Source: PeerJ. 2024 Oct 8;12:e18082. doi: 10.7717/peerj.18082 (PMC11468899; doi:10.7717/peerj.18082)
Supplement: Supplemental Information 9 — Normalized abundance of core ASVs is depicted in Figure 3. Core ASV names correspond to names in that figure. [file peerj-12-18082-s009.docx]

**Table S2. Details about core ASVs.** Normalized abundance of core ASVs is depicted in Figure 3. Core ASV names correspond to names in that figure.

| Core ASV name | Associated bivalve species | Sequence | Core ASV taxonomy |
| --- | --- | --- | --- |
| Core ASV 1 | *Crassostrea virginica, Macoma balthica, Ameritella mitchelli* | TACGTAGGGGGCGAACGTTGTTCGGAATCACTGGGCGTAAAGGGTGCGCAGGCTGCTCATCAAGTCAGTTGTGAAATTTCGTGGCTCAACCACGAAGCTGCTGCTGATACTGTTGGGCTAGAGTCATAGATAGAGGTAGCGGGAATTCCTGGTGTAGGAGTGAAATCTGTAGATATCAGGAGGAACACCAAGGGTGAAGACAAGCTACTGGGTAATGACTGACGCTGAGGCACGAAAGCTAGGGGAGCAAATGGG | Bacteria; Spirochaetota; Spirochaetia; Spirochaetales; Spirochaetaceae |
| Core ASV 2 | *Ischadium recurvum* | TACGTATGGGGCAAACGTTGTTCGGATTCACTGGGCGTAAAGAGTACGTAGGCTGTCGAGACAGTCAATGATCAAATCCCGTAGCTTAACTACGTGGTCGTTGTTGATACTACTCGACTAGAGTTTTATAGAGGTAGCGGGAATTCCTGGTGTAGGGGTGAAATCTGTTGATATCAGGAGGAACACCAAAGGCGAAGGCAAGCTACTGGGTAAAAACTGACGCTGAGGTACGAAAGCCAGGGGAGCAAATGGG | Bacteria; Spirochaetota; Spirochaetia; Spirochaetales; Spirochaetaceae |
| Core ASV 3 | *Macoma balthica, Ameritella mitchelli* | GACGAACCGTACAAACGTTACTCGGAATTACTGGGCTTAAAGGGTGCGTAGGCTGCGCGGAAAGTTGGGTGTGAAAGCCCTCGGCTCAACCGAGGAATTGCATCCAAAACTACCGTGCTGGAGGGAGACAGAGGTAAGCGGAACTCAAGGTGGAGCGGTGAAATGCGTTGATATCTTGAGGAACACCGGTGGCGAAAGCGGCTTACTGGGTCTCTTCTGACGCTGAGGCACGAAAGCTAAGGTAGCAAACGGG | Bacteria; Planctomycetota; Planctomycetes; Pirellulales; Pirellulacea |
| Core ASV 4 | *Ameritella mitchelli* | TACGGGAGTGGCAAGCGTTATCCGGAATTATTGGGCGTAAAGCGTCCGCAGGCGGTCTTGTAAGTCTGTTGTTAAAGCGTGGAGCTTAACTCCATTTCAGCAATGGAAACTGTAAGACTAGAGTGTGGTAGGGGCAGAGGGAATTCCCGGTGTAGCGGTGAAATGCGTAGATATCGGGAAGAACACCAGTGGCGAAGGCGCTCTGCTGGGCCATAACTGACGCTCATGGACGAAAGCCAGGGGAGCGAAAGGG | Bacteria; Cyanobacteria; Cyanobacteria; Synechococcales; Cyanobiaceae; Cyanobium PCC-6307 |
| Core ASV 5 | *Ameritella mitchelli* | TACGGGAGTGGCAAGCGTTATCCGGAATTATTGGGCGTAAAGCGTCCGCAGGCGGCCTTTTAAGTCTGTTGTTAAAGCGTGGAGCTTAACTCCATTTCAGCAATGGAAACTGGAAGGCTTGAGTGTGGTAGGGGCAGAGGGAATTCCCGGTGTAGCGGTGAAATGCGTAGATATCGGGAAGAACACCAGTGGCGAAGGCGCTCTGCTGGGCCATAACTGACGCTCATGGACGAAAGCCAGGGGAGCGAAAGGG | Bacteria; Cyanobacteria; Cyanobacteria; Synechococcales; Cyanobiaceae; Cyanobium PCC-6307 |
| Core ASV 6 | *Ameritella mitchelli* | GACGGAGGGGGCTAGCGTTGTTCGGAATTACTGGGCGTAAAGGGCGCGTAGGCGGATTAGTAAGTTGGGAGTGAAAGCCCGGGGCTTAACCTCGGAACTGCTTTCAAAACTGCTAGTCTTGAGTGAAGTAGGGGATGATGGAATTCCTAGTGTAGAGGTGAAATTCTTAGATATTAGGAGGAACACCGGTGGCGAAGGCGGTCATCTGGACTTCAACTGACGCTGAGGCGCGAAAGCGTGGGGAGCAAACAGG | Bacteria; Proteobacteria; Alphaproteobacteria; Rickettsiales; Rickettsiaceae |
