# Supplementary material for: Bivalve microbiomes are shaped by host species, size, parasite infection, and environment
Source: PeerJ. 2024 Oct 8;12:e18082. doi: 10.7717/peerj.18082 (PMC11468899; doi:10.7717/peerj.18082)
Supplement: Supplemental Information 11 [file peerj-12-18082-s011.docx]

### Table S4. PERMANOVA showing the effect of cohabitation and density on oyster microbiomes (2018 experiment)

| **Factor** | **Df** | **SumOfSqs** | **R2** | **F** | **Pr(>F)** |
| --- | --- | --- | --- | --- | --- |
| **Density** | 1 | 0.40 | 0.027 | 1.42 | 0.13 |
| **Cohabitation** | 1 | 0.28 | 0.019 | 1.01 | 0.36 |
| **Density:Cohabitation** | 1 | 0.20 | 0.014 | 0.72 | 0.69 |
| **Residual** | 54 | 13.69 | 0.94 | NA | NA |
| **Total** | 57 | 14.57 | 1 | NA | NA |
